# Dynamics of bed bug infestations and control under disclosure policies

**DOI:** 10.1073/pnas.1814647116

**Published:** 2019-03-04

**Authors:** Sherrie Xie, Alison L. Hill, Chris R. Rehmann, Michael Z. Levy

**Affiliations:** ^a^Department of Biostatistics, Epidemiology and Informatics, University of Pennsylvania, Philadelphia, PA 19104-6021;; ^b^Program for Evolutionary Dynamics, Harvard University, Cambridge, MA 02138;; ^c^Department of Civil, Construction, and Environmental Engineering, Iowa State University, Ames, IA 50011-1066

**Keywords:** bed bugs, disclosure, mathematical model

## Abstract

Bed bugs have reemerged in the United States and worldwide over recent decades, presenting a major challenge to both public health practitioners and housing authorities. A number of municipalities have proposed or initiated policies to stem the bed bug epidemic, but little guidance is available to evaluate them. One contentious policy is disclosure, whereby landlords are obligated to notify potential tenants of current or prior bed bug infestations. Aimed to protect tenants from leasing an infested rental unit, disclosure also creates a kind of quarantine, partially and temporarily removing infested units from the market. Here, we develop a mathematical model for the spread of bed bugs in a generalized rental market, calibrate it to parameters of bed bug dispersion and housing turnover, and use it to evaluate the costs and benefits of disclosure policies to landlords. We find disclosure to be an effective control policy to curb infestation prevalence. Over the short term (within 5 years), disclosure policies result in modest increases in cost to landlords, while over the long term, reductions of infestation prevalence lead, on average, to savings. These results are insensitive to different assumptions regarding the prevalence of infestation, rate of introduction of bed bugs from other municipalities, and the strength of the quarantine effect created by disclosure. Beyond its application to bed bugs, our model offers a framework to evaluate policies to curtail the spread of household pests and is appropriate for systems in which spillover effects result in highly nonlinear cost–benefit relationships.

## Significance

Bed bugs are household pests that bite humans and cause myriad medical, psychological, social, and economic problems. Infestation levels have resurged across the United States in recent decades, and cities and states are debating strategies to deal with them. Here, we introduce a mathematical model to study the spread of bed bugs and predict the costs and benefits of policies aimed at controlling them. In particular, we evaluate disclosure, a policy that requires landlords to notify potential tenants of recent infestations in a unit. While disclosure aims to protect individual tenants, our results suggest that these policies also reduce infestation prevalence market-wide. Disclosure results in some initial cost to landlords but leads to significant savings in the long term.

Bed bugs (*Cimex lectularius*) have reemerged in the United States and worldwide since the early 2000s (1–4). The prevalence of infestations in major US cities is high, although only poorly described. In 2014, the New York City Community Health Survey estimated the annual prevalence of bed bug infestations to be 5.1% city-wide and as high as 12% in some neighborhoods (5). Similarly, a door-to-door survey of bed bug infestations conducted in a Philadelphia census tract in 2013 found that 11.1% of respondents had recent bed bug infestations (6).

The health consequences of the current bed bug pandemic are inarguably enormous. Bed bugs inflict physical and psychological distress to those they bite and whose dwellings they infest, causing itching, rashes, allergies, sleep loss, anxiety, and other symptoms (7–9). In addition to these direct effects, bed bug infestations prevent homebound patients—especially senior citizens and disabled individuals—from receiving care, as many home-care providers are reluctant to enter infested houses (10, 11). Poisoning by insecticides inappropriately applied to combat bed bugs has caused at least one fatality and left scores acutely ill and countless more exposed, often unknowingly (12). Bed bugs are competent vectors of *Trypanosoma cruzi* (13, 14) and *Bartonella quintana* (15), the etiological agents of Chagas disease and trench fever, respectively. Whether or not bed bugs currently are, or will become, epidemiologically relevant in the transmission of infectious agents remains unknown.

The optimal political response to the bed bug epidemic has yet to be determined. Policies must balance the rights of tenants, landlords, and the public at large. Treatment of infestations with insecticide or heat-based interventions is generally paid for by individuals, not municipalities, and so policies strive to incentivize rapid and effective treatment while minimizing stigma, cost, and lost housing opportunities. An increasing number of US states and municipalities are responding to the rise in bed bug prevalence with disclosure policies, which require landlords to notify potential tenants of bed bug infestation histories. For example, in New York City, landlords are required to disclose the bed bug infestation and treatment histories of their units for the previous year to all tenants entering a lease agreement (16). Similar, though less stringent, versions of this policy have been passed in San Francisco (17); Mason City, Iowa (18); Connecticut (19); and Maine (20).

The primary aim of disclosure policies is to protect individuals from unknowingly leasing an infested rental unit. Nonetheless, these policies may have community-wide effects: Disclosure can decrease the desirability of infested units, thereby imposing a kind of partial quarantine that could decrease the prevalence of infestations on a city or even regional scale. The potential benefits of disclosure seemingly come at a cost, most of which would fall on the shoulders of landlords. These costs could manifest as both direct costs of pest-control treatment and lost rent due to increased vacancies and tenant turnover under disclosure. Disclosure laws have been actively contested by landlord organizations (21), and opponents fear they could stigmatize affected buildings and lower property values (22). But the infectious nature of bed bug infestations creates spillover effects that result in highly nonlinear cost–benefit relationships. If bed bug prevalence decreases following more proactive disclosure legislation, fewer treatments and less tenant turnover could increase the value of rental properties.

Here, we introduce a simple mathematical model to estimate the financial impact of bed bug disclosure policies on landlords over a range of time horizons. Our model adapts the traditional Susceptible–Infectious–Susceptible (SIS) framework originally developed to model infectious diseases (23) to capture housing-market dynamics. To understand the general behavior of this system, we derive the basic reproductive ratio, $R_{0}$, which provides insight on whether, over the long term, the prevalence of infestation is likely to trend toward zero or reach an endemic equilibrium. We use parameters derived from field studies and publicly available sources that are meant to approximately represent a single rent level in a major US city. In developing our model, we describe a shift from costs to savings associated with disclosure over a relatively short time horizon. In subsequent sensitivity analyses, we identify the key parameters driving our results.

## Methods

### A Susceptible-Infested-Susceptible Model for Bed Bug Transmission.

**Fig. 1.**
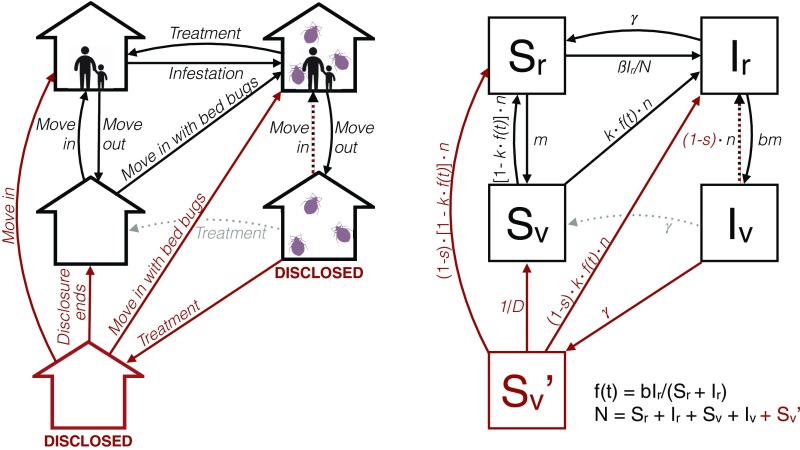
A mathematical model of bed bug spread with and without disclosure policies. *Left* shows the processes leading to transfer between rental unit types, while *Right* lists the state variable names and transition rates. Items in black are in the model regardless of whether disclosure policies are implemented; items in red are only in the model with disclosure; and items in gray are only in the model without disclosure. Rental units are classified by both their occupancy status and infestation status: susceptible-occupied ($S_{r}$), infested-occupied ($I_{r}$), susceptible-vacant ($S_{v}$), and infested-vacant ($I_{v}$). When disclosure is implemented, there is an additional susceptible-vacant-disclosed class ($S_{v}^{\prime }$), indicating a previous infestation which was treated. A detailed description of the transition rates is provided in the text. Parameter descriptions and values are in Table 1. The model equations are shown in Eqs. **1** and **2**.

Our model of bed bug transmission is an adaptation of the traditional SIS model (23). Here, the unit of infection is a rental unit, which we define to be any apartment unit, condominium, or other single-family dwelling that is rented or available for rent. Units free of bed bugs are considered susceptible (S), whereas infested (I) units harbor bed bugs. Due to the resilience of bed bug populations, we assume that a unit can move out of the I class only by receiving treatment. We consider a closed population of rental units of a constant size, N. In the basic model (Fig. 1), the S and I classes are further subclassified as renter-occupied (r) or vacant (v) depending on tenant occupancy, yielding a total of four classes: susceptible-occupied ($S_{r}$), infested-occupied ($I_{r}$), susceptible-vacant ($S_{v}$), and infested-vacant ($I_{v}$). The system of differential equations of the SIS model is as follows:

$$\begin{array}{cc} \frac{dS_{r}}{dt} & =-\beta S_{r}I_{r}/N+\gamma I_{r}+n\left(1-kf\left(t\right)\right)S_{v}-mS_{r}\\ \frac{dI_{r}}{dt} & =\beta S_{r}I_{r}/N+kf\left(t\right)nS_{v}+nI_{v}-\left(\gamma +bm\right)I_{r}\\ \frac{dS_{v}}{dt} & =mS_{r}+\gamma I_{v}-nS_{v}\\ \frac{dI_{v}}{dt} & =bmI_{r}-\left(\gamma +n\right)I_{v}\\ N & =S_{r}+I_{r}+S_{v}+I_{v}\\ f\left(t\right) & =\frac{bI_{r}}{S_{r}+bI_{r}}. \end{array}$$ [1]

Individuals move out of occupied units at a baseline rate m, therefore spending an average time 1/m in a given unit before moving. We assume that a bed bug infestation in an occupied unit increases the move-out rate by a factor b (b>1). New tenants move into vacant units (whether they are susceptible or infested) at a rate n, so that 1/n is the average time a unit remains vacant. Infested units are treated at rate γ, which implies that the average time for an infested unit to successfully initiate and complete treatment is 1/γ. Units can become newly infested by two mechanisms in our model: infectious transmission and relocation transmission. Both of these modes of transmission contribute to the system’s basic reproductive ratio, $R_{0}$, presented in *Results*. In infectious transmission, susceptible-occupied units become infested through importation of bugs on clothes, furniture, etc. at a rate assumed to be proportional to the infectivity, β, and the total prevalence of infested-occupied units in the population (i.e., $I_{r}/N$). In relocation transmission, individuals who have moved out of an infested unit may inadvertently bring bed bugs to the next unit they rent and seed a new infestation. We assume that the fraction of susceptible-vacant units that become infested via relocation transmission is the product of the presumed fraction of new occupants coming from previously infested apartments (f(t)) and the probability that these occupants establish bed bug populations in their new units upon arrival (k). f(t) is the ratio of the movement out of infested units ($bmI_{r}$) and the movement out of all rented units ($mS_{r}+bmI_{r}$), or $f\left(t\right)=bIr/\left(S_{r}+bI_{r}\right)$. Model parameters are listed and summarized in Table 1.

**Table 1.** Parameter point estimates and ranges used for sensitivity analysis

Parameters	Description	Best estimate	Range	Ref(s).
*p* (%)	Baseline prevalence	5	0.1–10	—
*β*	Infectivity	Fit	Fit	—
*s*	Renter selectivity	0.5	0.01–1	—
1/γ (mo)	Average duration of infestation	6	2–12	—
*k*	Probability of relocation transmission	0.3	0–1	—
*b*	Vacancy multiplier	1.3	1–5	—
*m* (y^–1^)	Move-out rate	0.5	—	(24)
*n* (y^–1^)	Move-in rate	6	—	(24, 25)
*D* (y)	Length of disclosure	1	—	(16)
Cost				
Treatment (*c*_*trt*_, $)	Average cost of successfully exterminating a bed bug infestation	1,225	—	(1)
Vacancy (*c*_*vac*_, $)	Average cost of a rental unit lacking tenants for 1 mo	1,000	—	(26)
Turnover (*c*_*tov*_, $)	Average cost of turning over a rental unit to new tenants	1,000	—	(27)

We make a few simplifying assumptions in assembling this model. We assume that rental units are homogeneous with respect to their market parameters (move-in and -out rates) and their bed bug transmission parameters (susceptibility to bed bugs, treatment rate, etc.). We also adopt the homogeneous mixing assumption held by many infectious disease models (23). Applied to the context of housing, this assumption means that any two rental units have an equal probability of “interacting” with each other, via in-person visits, exchange of objects, or turnover of tenants. We discuss the implications of relaxing these assumptions in the final section of *Results*.

### The Model in the Presence of Disclosure.

We expand the model to account for the effects of disclosure policies (Fig. 1). Under this model, units in the $I_{v}$ class are immediately disclosed and, upon treatment, move to a new susceptible-vacant-disclosed ($S_{v}^{\prime }$) class. The expanded model is as follows:

$$\begin{array}{cc} \frac{dS_{r}}{dt} & \;=\;-\beta S_{r}I_{r}/N+\gamma I_{r}+n\left(1-kf\left(t\right)\right)S_{v}+\left(1-s\right)n\left(1-kf\left(t\right)\right)S_{v}^{\prime }-mS_{r}\\ \frac{dI_{r}}{dt} & \;=\;\beta S_{r}I_{r}/N+nkf\left(t\right)S_{v}+\left(1-s\right)nkf\left(t\right)S_{v}^{\prime }+\left(1-s\right)nI_{v}-\left(\gamma +bm\right)I_{r}\\ \frac{dS_{v}}{dt} & \;=\;mS_{r}+\left(1/D\right)S_{v}^{\prime }-nS_{v}\\ \frac{dI_{v}}{dt} & \;=\;bmI_{r}-\left(\gamma +\left(1-s\right)n\right)I_{v}\\ \frac{dS_{v}^{\prime }}{dt} & \;=\;\gamma I_{v}-\left(\left(1-s\right)n+1/D\right)S_{v}^{\prime }\\ N & \;=\;S_{r}+I_{r}+S_{v}+I_{v}+S_{v}^{\prime }\\ f\left(t\right) & \;=\;\frac{bI_{r}}{S_{r}+bI_{r}}. \end{array}$$ [2]

We assume all disclosed units are less desirable to potential tenants proportional to a renter-selectivity parameter (s). Units in the $I_{v}$ and $S_{v}^{\prime }$ class might be thought to be in a “leaky quarantine,” the strength of which is determined by s. When s=1, no currently ($I_{v}$) or recently ($S_{v}^{\prime }$) infested units are rented out, meaning that disclosure results in a full quarantine. At the other extreme, when tenants do not change their renting behavior based on a unit’s disclosure status (s=0), the model reduces to the model in the absence of disclosure. We assume landlords comply fully with disclosure and units in class $S_{v}^{\prime }$ return to class $S_{v}$ at rate 1/D, where D represents the mandated disclosure period. Although in reality disclosure is of a fixed length, we have modeled it as a continuous transition for simplicity. In *SI Appendix*, we show that relaxing this assumption does not impact our conclusions.

### Estimating Disclosure Costs.

Our primary outcome of interest is the change in expected cost due to disclosure, which we define as the difference in cost to landlords in the presence of disclosure compared to the absence of disclosure. In the context of a rental market with bed bugs endemic, cost can take the following forms: bed bug treatment costs, rental turnover costs, and opportunity costs due to vacancy. Bed bug treatment costs are the expenses associated with the extermination or attempted extermination of bed bugs and include fees to pest-control companies and contractors. Turnover costs include the expenses involved in repairing, advertising, and showing units to prospective tenants. Opportunity costs due to vacancy are incurred anytime a rental unit lacks tenants and are equal to the rental price for that unit. Hereafter, we will use the term “cost” to refer to total additional cost to landlords that result from disclosure. Similarly, we will use the terms “treatment cost,” “turnover cost,” and “vacancy cost” to refer to these component costs with respect to disclosure (i.e., the difference of each component in the presence and absence of disclosure).

The average per-unit number of bed bug treatments occurring in a given year is equal to the number of transitions from infested to susceptible classes for that year divided by the number of rental units in the system *N*, and the average number of turnover events is equal to the number of transitions from vacant to occupied classes divided by *N*. Similarly, the average time that each unit is vacant is equal to the total unit-time spent in vacant classes divided by *N*. Then, if $c_{trt}$, $c_{tov}$, and $c_{vac}$ are constants equal to the average ancillary cost of bed bug treatment, average cost of moving, and average cost of untreated infestation, respectively, the component costs of disclosure from the perspective of renters can be expressed for a given year Y by the following:

$$\begin{array}{cc} \text{Treatment cost}\;=\; & \frac{c_{trt}}{N}\left({\left.\int_{Y}^{\left(Y+1\right)}\left(\gamma I_{r}+\gamma I_{v}\right)dt\right|}_{s=s}-{\left.\int_{Y}^{\left(Y+1\right)}\left(\gamma I_{r}+\gamma I_{v}\right)dt\right|}_{s=0}\right)\\ \text{Turnover cost}\;=\; & \frac{c_{tov}}{N}\left({\left.\int_{Y}^{\left(Y+1\right)}\left(nS_{v}+n\left(1-s\right)S_{v}^{\prime }+n\left(1-d\right)I_{v}\right)dt\right|}_{s=s}\right.\\ & -\left.{\left.\int_{Y}^{\left(Y+1\right)}\left(nS_{v}+n\left(1-s\right)S_{v}^{\prime }+n\left(1-d\right)I_{v}\right)dt\right|}_{s=0}\right)\\ \text{Vacancy cost}\;=\; & 12\frac{c_{vac}}{N}\left({\left.\int_{Y}^{\left(Y+1\right)}\left(S_{v}+S_{v}^{\prime }+I_{v}\right)dt\right|}_{s=s}\right.\\ & -\left.{\left.\int_{Y}^{\left(Y+1\right)}\left(S_{v}+S_{v}^{\prime }+I_{v}\right)dt\right|}_{s=0}\right). \end{array}$$ [3]

Above, each component cost is calculated as the difference in the average quantity of treatment, turnover, and vacancy in the presence vs. in the absence of disclosure multiplied by the cost constant. Cost can then be calculated as the sum of the treatment, turnover, and vacancy costs.

### Model Implementation and Parameter Estimates.

We ran the model with a total (N) of 1,000 units (although our results are insensitive to population size). The average cost of bed bug treatment was set to equal $1,225, the median cost of bed bug treatment for single-family homes reported by a national survey of pest-management professionals in 2015 (1). The average cost of turnover was set equal to $1,000, a figure that has been cited on property-management blogs (24). The average monthly rent (and monthly opportunity cost due to vacancy) was set to equal $1,000, roughly the national median reported by the American Community Survey for 2017 (25).

Initial conditions for the start of each simulation were the equilibrium values for the same system in the absence of disclosure, assuming that the overall baseline prevalence of infestation, $\left(I_{r}+I_{v}\right)/N$, was p:

$$\begin{array}{cc} S_{r}* & \;=\;\frac{N}{m+n}\left(\left(1-p\right)n-pm\frac{b\gamma }{bm+\gamma +n}\right)\\ I_{r}* & \;=\;Np\frac{\gamma +n}{bm+\gamma +n}\\ S_{v}* & \;=\;N\frac{m}{m+n}\left(\left(1-p\right)+p\frac{b\gamma }{bm+\gamma +n}\right)\\ I_{v}* & \;=\;Np\frac{bm}{bm+\gamma +n}. \end{array}$$ [4]

Estimated values or ranges for parameters are reported in Table 1. Move-in and -out rates were estimated according to data from the US Census Bureau Housing Vacancy Survey for 2017 and the 2017 National Apartment Association Survey of Operating Income and Expenses in Rental Apartment Communities (26, 27). The move-out rate, m, was estimated based on the average frequency of moves (once every 2 y). To estimate the move-in rate (n), we calculated the percent of units that would be vacant in our model at baseline [percent vacant = $\left(S_{v}+I_{v}\right)/N$ in Eq. **4**, ≈m/(n+m) when prevalence is low] and chose a move-in rate so this matched the national average of 7% rental vacancy. The length of disclosure *D* was set to equal 1 y, which is equivalent to the length mandated by New York City (16). The infectivity β cannot be observed directly; we calculated it by solving for the value that would yield a given baseline prevalence p, which is more easily observed and interpreted:

$$\beta =\frac{N}{S_{r}*I_{r}*}\left(\left(\gamma +bm\right)I_{r}*-\frac{kbI_{r}*}{S_{r}*+bI_{r}*}nS_{v}*-nI_{v}*\right),$$ [5]

where $S_{r}*$, $I_{r}*$, $S_{v}*$, and $I_{v}*$ are given in Eq. **4**.

Some parameters, including the average duration of infestation (1/γ), probability of relocation transmission (*k*), and vacancy multiplier (*b*), could not be estimated from available data; they were thus assigned realistic point values and assessed over ranges of values in subsequent sensitivity analyses. No data exist on the duration of bed bug infestations, although there are several factors impeding timely treatment. Recent genetic analyses suggest that infestations are founded by small populations consisting of few individuals or even a single mated female (28, 29), and reactions to bites are nonspecific and often misdiagnosed (30, 31), both of which retard detection by tenants and landlords. Even detection by pest-management professionals, which occurs by visual inspection and is sometimes aided by trained canines, has imperfect sensitivity and specificity (31). Moreover, treatment failure is common, even after multiple visits (32–34). Due to the challenges involved in bed bug detection and treatment, the average duration of infestation was estimated to equal 6 mo; sensitivity analysis evaluated how results changed if 1/γ were as brief as 2 mo or as long as 1 y.

There are anecdotal reports of tenants moving out of apartments prematurely due to bed bug infestations (35, 36), but data that can be used to estimate the factor by which infestation increases move-out rate are lacking. We chose a relatively conservative estimate for *b* (1.3, where move-out is assumed to be 30% greater in infested units relative to noninfested units) and found in subsequent sensitivity analyses that higher values of *b* led to even greater prevalence reduction and cost savings over the long term (*SI Appendix*, Fig. S9). Because bed bugs find harborage in furniture and clothing and much of their long-range dispersal is believed to be human-mediated (29), we reasoned that relocation transmission (whereby individuals moving out of an infested unit inadvertently bring bed bugs that seed a new infestation in their next unit) occurs, although at an unknown rate. Given the lack of data with which to estimate the probability of relocation transmission *k*, we set it to an intermediate value (0.3). Sensitivity analyses determined results to be robust to changes in *k* across its full range of possible values (*SI Appendix*, Figs. S6 and S7).

The model was coded and run in R using a differential equation solver in the *deSolve* library (37), and results were reported after each 1-y interval. An R Shiny web application allowing users to simulate our model themselves under alternate parameter values and visualize the output is available at https://bedbugdisclosure.shinyapps.io/shinyapp/. All analyses and figures presented in *Results* and *SI Appendix* can be reproduced by using code we have made available at https://github.com/sherriexie/bedbugdisclosure.

## Results

### Effects of Disclosure on Cost and Prevalence.

**Fig. 2.**
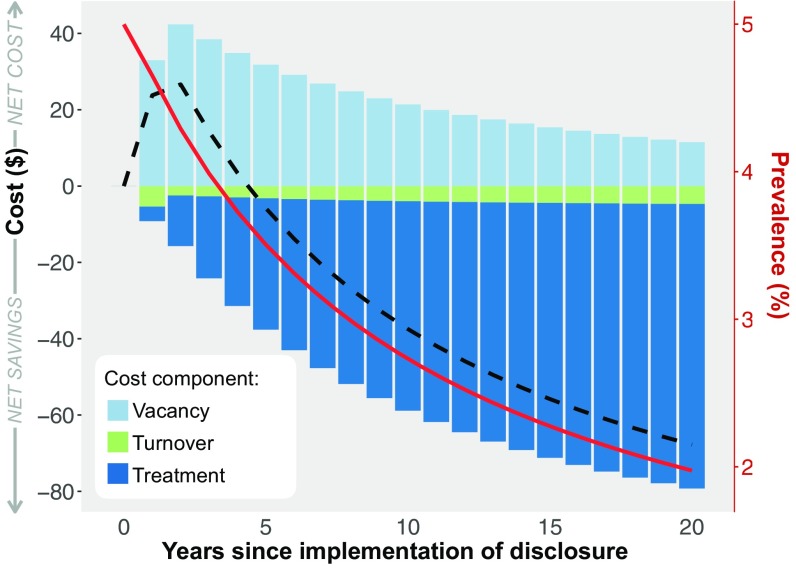
Impact of disclosure on bed bug prevalence and cost over time. Cost to landlords, defined as the difference in average, per-unit cost in the presence of disclosure compared with no disclosure, is shown by the dashed black line. The components of cost are shown as bars representing averages over 1-y periods and are broken down into cost due to unrented vacant units (“vacancy”), cost due to treating infested units (“treatment”), and cost from moving tenants into vacant units (“turnover”). The overall prevalence of infestation in the population is shown by the solid, red line. The model was run by assuming that before the implementation of disclosure, the baseline prevalence of infestations was at a steady-state value of 5%. We assumed that disclosure discouraged but did not prevent rental of disclosed units (s=0.5). Other parameter values are shown in Table 1, and results for additional parameter values are shown in *SI Appendix*.

Using our model and our best estimated parameter values (Table 1), we evaluated the impact of a newly implemented disclosure policy on the prevalence of bed bugs and the cost to landlords (Fig. 2). The cost of disclosure is high initially—reaching $25 per unit on the market after 2 y—but it decreases steadily, so that by year 5, landlords experience savings. The trends in total cost can be understood by examining the cost components. While turnover cost remains relatively constant and minimal, vacancy and treatment costs vary over time. Vacancy cost escalates directly after the implementation of disclosure, as disclosure makes infested and recently infested units less appealing to potential tenants. Meanwhile, this pseudo-“quarantine” of infested and recently infested units causes a steady decrease in prevalence (Fig. 2). Accordingly, the cost of treatment starts slightly negative—reflecting a cost savings, and these savings increase over time as prevalence continues to decline. Because bed bug infestations increase vacancy due to the larger move-out rate (b>1), the decline in prevalence also mitigates the effect of disclosure on vacancy; vacancy cost— although high initially—decreases over time. The net effect is that cost is high when disclosure is first introduced but quickly converts to savings that subsequently increase with time.

**Fig. 3.**
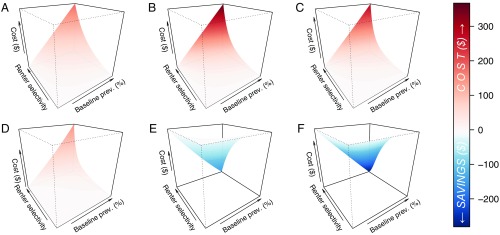
Total per-unit cost due to disclosure over time, as a function of the baseline prevalence (prev.) and renter selectivity. Results are presented for years 1 (*A*), 2 (*B*), 3 (*C*), 4 (*D*), 5 (*E*), and 20 (*F*) after the implementation of a disclosure policy. Cost is calculated as the sum of the total cost in the population due to vacancy, treatment, and tenant turnover, for the given 1-y interval, averaged over the total rental units. Red indicates situations where the cost to landlords is higher due to disclosure, whereas blue indicates situations where costs have decreased from baseline (savings). Baseline prevalence (p) ranges from 0.1 to 10%, and renter selectivity (s) ranges from 0.01 to 1. To see the dependence of the cost components on these parameters, refer to *SI Appendix*, Fig. S1. An animation showing the dependence of cost on p and s over the initial 20 y of disclosure is available at https://bedbugdisclosure.shinyapps.io/shinyapp/.

**Fig. 4.**
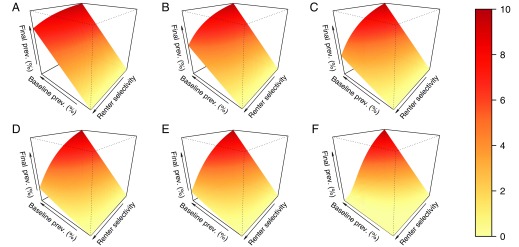
Prevalence of bed bug infestations over time after implementation of disclosure, as a function of the baseline prevalence (prev.) and renter selectivity. Results are presented as the year-end (“final”) prevalence for years 1 (*A*), 2 (*B*), 3 (*C*), 4 (*D*), 5 (*E*), and 20 (*F*) after the implementation of a disclosure policy. Baseline prevalence (*p*) ranges from 0.1 to 10%, and renter selectivity (s) ranges from 0.01 to 1. The origin is the bottom vertex farthest to the right; therefore, the baseline case of no disclosure is represented by the line of slope 1 in the back face on the right. To see these plots for prevalence reduction, refer to *SI Appendix*, Fig. S2. An animation showing the dependence of year-end prevalence on p and s he initial twenty years of disclosure is available at https://bedbugdisclosure.shinyapps.io/shinyapp/.

We examined in more detail how the predicted impact of disclosure policies depends on two parameter values that may vary between municipalities and are difficult to estimate: the baseline prevalence and the renter selectivity s (Figs. 3 and 4 and *SI Appendix*, Figs. S1 and S2). In all cases, year 5 marks the approximate turning point where vacancy costs are offset by savings from decreased treatment and total cost begins to dip below zero (Fig. 3*E*). In the initial years of disclosure, costs are greater if baseline prevalence is higher, and they are greatest when both the baseline prevalence and renter selectivity are high (Fig. 3 *A*–*D*). The initial effect of disclosure policies under parameter regimes where both baseline prevalence and renter selectivity are high is to increase vacant units and the associated vacancy costs. In later years, the trend between cost and baseline prevalence actually reverses, and higher baseline prevalence results in increased savings. The same combination of high baseline prevalence and high renter selectivity that resulted in the greatest cost during the initial years of disclosure results in the greatest savings in later years. If we discount costs and savings that occur in later years (using methods detailed in *SI Appendix*), the results are similar, although the eventual savings decrease by an amount that is commensurate with the discount rate (*SI Appendix*, Fig. S3).

The greater cost savings accrued in later years are mediated by the effect of disclosure on the overall bed bug infestation prevalence (Fig. 4). Reductions in prevalence are seen as long as tenants show any selectivity in favor of units with no disclosed bed bug history compared with units with a disclosed bed bug history (s>0) but are more extreme for greater renter selectivity (s→1). This result holds at all times, and in some cases, we predict that prevalence can be driven to zero (bed bugs eliminated from the population). Overall, this analysis suggests that situations with unfavorable initial costs may be the same situations which lead to greater savings and prevalence reduction in the long run.

In addition to estimating disclosure costs for landlords, we also assessed the economic impact of disclosure on renters via methods outlined in *SI Appendix*. Disclosure policies are aimed at protecting tenants, and, as expected, we found this group to benefit financially from disclosure. Unlike landlords, who do not experience a net savings until later years, renters immediately benefit, with savings that grow over time as bed bug prevalence falls (*SI Appendix*, Fig. S4). In simulations with higher initial bed bug prevalence or higher renter selectivity (s), the savings in any given year are higher (*SI Appendix*, Fig. S5).

### Analytic Results and Threshold Behavior.

Similar to classic SIS infection models, our model of bed bug spread and control has two possible long-term outcomes: persistence of infection at an endemic equilibrium or decline of infestation levels toward zero. For any particular parameter set, one and only one of these outcomes represents a stable steady state of the dynamical system. We used the next-generation matrix method (38, 39) to calculate the basic reproductive ratio $R_{0}$ for our model as

$$R_{0}=\left(\frac{n}{n+m}\beta +kbm\right){\left[\gamma +bm\frac{\gamma }{n\left(1-s\right)+\gamma }\right]}^{-1}.$$ [6]

$R_{0}$ describes the average number of secondary infestations caused by the introduction of a single infested unit into a market of otherwise susceptible units. In addition, it determines the stability of the two possible equilibria for this model: Infestation persists as long as $R_{0}>1$ and declines to zero for $R_{0}<1$. In the absence of infestation (Eq. **4** with p=0), a fraction $S_{r}^{*}\left(0\right)/N=n/\left(n+m\right)$ of units are rented, and $S_{v}^{*}\left(0\right)/N=m/\left(n+m\right)$ are vacant. The first part of $R_{0}$ (within the parentheses) describes the initial spread of infestations by two independent routes: infectious transmission of already rented units [$\beta S_{r}^{*}\left(0\right)$] and relocation transmission of previously vacant units [$nkf\left(0\right)S_{v}^{*}\left(0\right)=kbm$]. The second part is the average time that an infested unit stays infested before being treated (either while still occupied or after being vacated). Disclosure reduces this infestation time by discouraging renters from moving into infested vacant apartments, preventing these units from contributing to interhousehold transmission.

For our baseline parameter values (Table 1 and β calibrated to give p=5% steady-state prevalence), $R_{0}$ is near 1: In the absence of disclosure (s=0), $R_{0}=1.05$, and it decreases to 0.88 as the renter selectivity increases to 1. $R_{0}$ is dominated by infectious transmission, while relocation transmission plays a more minor role (contributing ∼8%). Note that the length of the disclosure period does not directly influence the value of $R_{0}$, and hence will not affect the persistence of infestation. Disclosure policies will have more impact on the value of $R_{0}$ when there is more apartment turnover (higher m and n) and when tenants are effectively turned off from disclosed rental units (s→1).

The functional form of $R_{0}$ also suggests that other additional policies could have a greater impact on reducing prevalence than disclosure alone. Policies that incentivize the rapid identification and treatment of bed bug infestations (increasing γ) are likely to have the greatest effect in terms of stemming the epidemic. Disclosure policies, if enacted well, may be among these, as landlords would likely wish to retain tenants in infested units, and treat these effectively, to avoid the requirement of disclosing infestations to future tenants.

### Sensitivity of Results to Parameter Estimates.

We analyzed the sensitivity of results to uncertainty in the estimates for the probability of relocation transmission (*k*), average duration of infestation (1/γ), and vacancy multiplier (*b*). We recalculated the cost of disclosure while varying each parameter across the range given in Table 1 and holding all other parameters constant at their estimated values. Estimated cost and final prevalence were insensitive to k (*SI Appendix*, Figs. S6 and S7), more sensitive to 1/γ (*SI Appendix*, Fig. S8), and most sensitive to b (*SI Appendix*, Fig. S9). For example, doubling the baseline value of k led to little change, even if we assume that b is much higher (5 vs. 1.3) so that relocation transmission is responsible for ∼50% of total transmission. Doubling the length of infestation 1/γ yielded similar prevalence at year 20 and savings that were 60% larger. However, doubling the amount by which infestation increases move-out rate (b) reduced prevalence at year 20 by a factor of 2 and increased savings by a factor of 2.7; this result suggests that the predictions at the baseline value of b are conservative.

These sensitivity results should not be interpreted as contradicting a key analytical result above: that policies which would change the treatment rate would have a large impact on bed bug prevalence. Our model was implemented by calibrating the β parameter to a value that would give the desired baseline prevalence after all other parameter values (including k, γ, and b) had been assigned. Thus, our sensitivity analysis explored how our estimates of disclosure cost are affected by inaccuracies in these input parameters given a fixed prevalence.

The values of the cost parameters used to calculate vacancy, treatment, and turnover costs (Table 1) can vary by geographic region and can influence the impact of disclosure policies on landlords. Overall, in regions with lower monthly rents, but no difference in treatment or turnover costs, the initial costs of disclosure for landlords will be lower and will convert to savings more quickly. The converse is true for regions with higher rents. Even rents at the 95th percentile of those reported in the American Community Survey (∼$2,000) lead to savings within ∼7 y (40). Results for alternative costs can be explored with our online tool: https://bedbugdisclosure.shinyapps.io/shinyapp/.

### Impact of Intermarket Migration.

To determine how a rental market with legislated disclosure policies might be impacted by surrounding markets that do not adopt such policies, we considered a model that relaxes the assumption of a closed population and includes immigration of new tenants from external markets with a stable bed bug endemic. This model, outlined in *SI Appendix*, assumes that a fraction, i, of new tenants moving into vacant units come from external markets that have a net bed bug prevalence e. First, we assumed that the prevalence of bed bug infestation in the external market (e) was 5% and examined the effect of disclosure on cost and bed bug prevalence ranging the external tenant fraction (*i*, the proportion of new rentals that are taken by external vs. internal tenants) from 0 to 40%. Second, we assumed that 20% of new rentals were by tenants from an external market (i=0.2) and then evaluated cost and prevalence for a range of possible levels of infestation prevalence in these immigrant tenants (e from 5 to 20%). As expected, we found that migration into the system decreased the eventual savings caused by disclosure policies but that these savings were still apparent after approximately 5 y and significant after 10 y in all cases (*SI Appendix*, Fig. S10 *A* and *C*). Similarly, migration from regions with stable infestation levels dampened the decline in prevalence under disclosure policies, particularly when bed bug prevalence in the external market was much greater than the baseline prevalence of the system. For instance, prevalence declined to only 3.7% after 10 y when 20% of new tenants were immigrants from an external market with 20% prevalence, compared with 2.7% in the reference case with no immigration (*SI Appendix*, Fig. S10*D*). When the external prevalence is comparable to the baseline prevalence of the system (*e* = 5%), intermarket migration had less impact on prevalence decline, even with a large fraction of immigrant tenants (*i* = 40%; *SI Appendix*, Fig. S10*B*).

### Impact of Disclosure in a Structured Population.

Our model so far has assumed that the population of rental units susceptible to infestation is uniform and well-mixed. However, it is also possible that certain subsets of the population play an outsized role in sustaining the epidemic, perhaps due to inability to afford prompt treatment or more frequent movement between apartments. Infestation prevalence in such hypothetical subpopulations might be more difficult to control with disclosure or other policies, and the cost and benefit estimates could be different. Constructing a realistically heterogeneous model of bed bug transmission and control is difficult, given the very limited surveillance data, so we created simple two-population models to evaluate some worst-case scenarios using methods detailed in *SI Appendix*. In each model, a small, high-prevalence subpopulation is completely disconnected from a larger, lower-prevalence population, and the high-prevalence subpopulation can sustain higher infestation rates by having a lower treatment rate γ, a higher move-out rate m, or a higher “aversion to bed bugs” (higher b and k). We found, in each scenario, that landlord costs remained positive and did not convert to savings in the high-prevalence subpopulation. These results show that if infestation is extremely concentrated in one segment of the population, landlords serving that population will bear most of the short-term costs and reap most of the long-term benefits of disclosure policies—but that sometimes savings never occur. This major difference occurs because effective $R_{0}$ values may be much higher in these high-prevalence subpopulations, meaning that control measures need to be much more severe to significantly reduce infestation levels. Disclosure policies would likely need to be combined with other interventions, such as decreasing time to treatment in high-prevalence subpopulations.

## Discussion

The spread of bed bugs is a growing concern in cities around the world, and despite the obvious similarity to infectious diseases and the need for evidence-based control policies, bed bugs have received little attention from mathematical models (41). In this work, we introduce a simple model that incorporates the major defining features of bed bug spread. We track the dynamics of both the housing market and infestation prevalence, since the interaction between these two processes, which occur on similar timescales, is a key feature of bed bug outbreaks. Tenant turnover contributes to the spread of infestation between rental units, and infestations can lead to increased rates of vacating apartments and reluctance to move into units with a history of infestation. Our model explicitly considers two general modes by which bed bugs can spread between units—either by tagging along with tenants who leave infested units for new ones or by importation into bug-free occupied units—and provides a framework to estimate the relative contribution of these processes based on individual parameters describing components of human or bed bug behavior. This model can be used as a framework for evaluating proposed control measures.

In response to the recent resurgence of bed bug infestations, some states and municipalities have adopted bed bug disclosure laws that require landlords to disclose the infestation histories of their units to all potential tenants [New York City (16)] or to tenants upon request [San Francisco (17), Mason City (18), Maine (20), and Connecticut (19)]. These policies seek to protect tenants, but some fear that such measures would impose costs that would unfairly punish landlords. We used our model of bed bug transmission to evaluate the potential impact of disclosure policies on bed bug prevalence and cost to property owners. Overall, we found that the financial impact of disclosure to landlords varies over time: beginning as a net cost, but peaking quickly and subsequently falling continuously. Contrary to fears that disclosure policies would create significant economic burden to landlords, we predict that, in many scenarios, they are likely to result in significant savings over relatively short time horizons.

Our results suggest that the magnitude of the initial cost, and the eventual savings, are driven by two key factors: the prevalence of bed bug infestations before disclosure and renter selectivity. When prevalence is relatively high (closer to ∼10% vs. ∼1–2%), implementation of disclosure creates more vacant units initially but eventually leads to significant savings as prevalence drops. Renter selectivity is a theoretical value that would depend, in part, on knowledge and attitudes toward bed bugs and also, in part, on the supply and demand for rental units in a regional market. In cities with an abundance of rental units, renter selectivity might be higher because in these “renters’ markets,” renters can afford to be more choosy. On the other hand, in cities like New York City and San Francisco, where there is a relative housing shortage and demand for rental units is high (42, 43), renter selectivity is expected to be low. It is perhaps not a coincidence, then, that it is in these cities that bed bug disclosure has been legislated in some form, since the immediate economic risk to landlords is likely to be low.

Although our work focused primarily on the financial impact of disclosure on landlords, we also found disclosure to benefit tenants. Infestations can be a costly ordeal for tenants, even when landlords bear sole financial responsibility for extermination. Costs to tenants can include replacing furniture and so-called do-it-yourself treatments, which may result in property or bodily damage (12, 35, 44). Frustration with the inability to eliminate bed bug infestations can lead tenants to vacate their homes, sometimes breaking their lease to do so (36). The decrease in prevalence that is likely to result from well-enacted disclosure policies would intuitively benefit tenants, whom we expect to experience savings from the first year of policy implementation. Moreover, these benefits are likely to extend beyond the rental market to private homeowners, who may acquire infestations from renters, and local governments, some of which are very large landlords of public housing (45).

We found, using reasonable parameter regimes and assuming a baseline steady-state prevalence of <10%, that the basic reproductive ratio, $R_{0}$, of bed bug infestations is close to 1. Consequently, small perturbations to the system, due to disclosure or other policies, can push bed bug populations toward local elimination (since $R_{0}>1$ is needed for persistence). Despite significant uncertainty surrounding several model parameters, there is good reason to believe that this finding is accurate. Bed bug populations were easily eliminated on a grand scale following the availability of dichlorodiphenyltrichloroethane (DDT) and other synthetic insecticides in a way that other household pests, such as cockroaches, were not (46). If $R_{0}$ of bed bug infestations were much greater than one, it is highly unlikely that their populations would have crashed so dramatically. It is equally unlikely that it would have taken so long for bed bugs to reemerge following their development of resistance to DDT and pyrethroid insecticides [which was reported as early as 1948 (47)].

An important assumption we make in calculating β, and hence $R_{0}$, is that the prevalence of bed bug infestations reported from field studies represents a system at or near equilibrium. While this assumption is supported by general patterns present in internet search patterns (*SI Appendix*, Fig. S11), these have their own complex dynamics that may not directly mirror the prevalence of infestations (48). If bed bug infestations are indeed continuing to climb, and we have underestimated the infectivity, the break-even timepoint, at which the cost of disclosure equals the savings due to decreased prevalence, would be delayed. If the true equilibrium prevalence of infestation exceeds a threshold (∼16% based on our best estimates of the parameters), the break-even point could be delayed indefinitely (*SI Appendix*, Fig. S12). However, savings can be recovered if disclosure is more effective at averting tenants from infested units than we estimated (s→1) or if disclosure policies also improve treatment rates.

Due to the relative dearth of data on bed bug infestations, our model was formulated under a few simplifying assumptions, and our results should be interpreted in the context of these limitations. Our model does not incorporate elasticities that are likely to exist in the rental-housing market. Landlords might prefer to lower the rent of disclosed units rather than let them sit vacant, and not allowing prices to respond to disclosure may lead us to overestimate vacancy costs and, potentially, the decrease in prevalence due to the quarantine effect. Our model is most relevant to a single segment of a rental market in which units, landlords, and tenants are expected to be relatively similar. Results from our metapopulation models, which considered two hypothetical subpopulations that exist in isolation, suggest that disparities in the populations of interest that are not accounted for in our mass-action model could lead us to overestimate the benefits of disclosure; however, some benefits are likely to be recovered with more realistic levels of intermediate mixing between groups. We did not consider more complex metapopulation or network structures beyond our two-population models, and it is unclear before parametrizing such models how their results might diverge from those obtained from our mass-action model (49).

While our model included a single value for the treatment rate (γ), it was able to at least partially account for variability in treatment time that could result from some infestations being intrinsically more difficult to detect or treat; this property follows from the formulation of our model as a system of ordinary differential equations, which makes the implicit assumption that treatment times are exponentially distributed (and thus have a long tail). However, our model does not include temporal dynamics and feedback effects on γ. Disclosure policies require the disclosure of treatment along with infestation histories and would likely put pressure on landlords to treat vacant units before showing them to potential tenants. Not capturing these changes in γ as landlords respond to disclosure may lead us to underestimate the benefits of disclosure. On the other hand, our model does not and cannot anticipate the evolution of additional insecticide resistance in bed bug populations, which could have enormous effects on γ and the future of the epidemic as a whole. Additional model limitations, along with possible extensions, are presented in *SI Appendix*.

Despite recent advances in pest-management strategies (34, 50) and improvements to urban housing, bed bugs have reemerged as a household pest and public health concern. Our model provides a first step toward evidence-based prospective analysis of policies to control the spread of bed bugs. Our results show that bed bug control is a classic collective action problem: Individual landlords bear the initial costs of disclosure policies, but after a few years, both landlords and tenants will benefit from the reduction in prevalence of infestations. Additionally, we show that while bed bugs are extremely difficult to eliminate from homes, they are likely to be less difficult to control in cities. We predict that, on average, a single infested residence infests little more than one additional residence ($R_{0}≳1$), whether by infectious or relocation transmission. Consequently, rational and enforced policies have great potential to stem the bed bug epidemic.

## Supplementary Material

Supplementary File
