# Supplementary material for: Dynamics of bed bug infestations and control under disclosure policies
Source: Proc Natl Acad Sci U S A. 2019 Mar 4;116(13):6473–81. doi: 10.1073/pnas.1814647116 (PMC6442569; doi:10.1073/pnas.1814647116)
Supplement: Supplementary File [file pnas.1814647116.sapp.pdf]

Supporting Information

Supplementary Text

Approximating a discrete disclosure period

The models presented in the main text (Eqs. 1 and 2) implicitly assume a continuous rate of transition of units into and out of specific infestation/rental states. This type of approximation is ubiquitous in population dynamics, and generally results in very good descriptions of the mean behavior of the system when population sizes are relatively large, transitions happen relatively quickly, and dynamics are not synchronized. However, there is one process in our model for which this formulation could be problematic: the disclosure period. If disclosed units were never rented ($s = 1$), then units would exit the disclosed state (S'_v) continuously at a rate $1/D$. This implies that the precise time that any individual unit spends in the disclosed state is a random variable τ with an exponential distribution, $p(\tau) = (1/D)e^{-\tau/D}$. However, real-life disclosure policies mandate a precise length for the disclosure period, not a distribution of lengths. We were interested in examining whether this assumption influenced any of our predictions about the impact of disclosure policies.

To evaluate discrete disclosure periods while maintaining our ordinary differential equation framework, we leveraged the fact that the sum of exponentially-distributed random variables – each with the same rate – results in a gamma-distributed variable with predictable parameters [S1, S2]. We assume there are n_D distinct disclosed states that a unit can be in ($S'_{v,i}$, for $i = 1...n_d$). Treated units enter the first disclosed state ($S'_{v,1}$), and units progress between each sequential disclosed state at rate n_D/D. Units in the last disclosed state transition to the non-disclosed susceptible vacant state S_v at rate n_D/D. Thus, if disclosed units are never rented, the distribution of times τ spent in the disclosed state follows the gamma distribution $p(\tau) = (n_D/D)^n \tau^{n_D-1} e^{-n\tau/D}/(n-1)!$. The average time spent disclosed is $\langle\tau\rangle = D$, as in the original model, and the standard deviation is $\sigma_\tau = D/\sqrt{n_D}$. As the number of disclosed compartments is increased ($n_D \to \infty$), the disclosure period becomes approximately fixed in length ($\sigma_\tau \to 0$). When disclosed units can be rented ($s \neq 1$), then the rate of exit via rental is equal between all disclosed states (and the average time spent disclosed will always be less than D). The equations for this model then become:

$$
\begin{aligned}
\frac{dS_r}{dt} &= -\beta S_r I_r/N + \gamma I_r + n(1-kf(t))S_v + (1-s)n(1-kf(t))\sum_{i=1}^{n_D} S'_{v,i} - mS_r \\
\frac{dI_r}{dt} &= \beta S_r I_r/N + nkf(t)S_v + (1-s)nkf(t)\sum_{i=1}^{n_D} S'_{v,i} + (1-s)nI_v - (\gamma + bm)I_r \\
\frac{dS_v}{dt} &= mS_r + (n_D/D)S'_{v,n_D} - nS_v \\
\frac{dI_v}{dt} &= bmI_r - (\gamma + (1-s)n)I_v \\
\frac{dS'_{v,1}}{dt} &= \gamma I_v - ((1-s)n + n_D/D)S'_{v,1} \\
\frac{dS'_{v,i}}{dt} &= (n_D/D)S'_{v,i-1} - ((1-s)n + n_D/D)S'_{v,i} \qquad 2 \leq i \leq n_D \\
N &= S_r + I_r + S_v + I_v + \sum_{i=1}^{n_D} S'_v \\
f(t) &= \frac{bI_r}{S_r + bI_r}
\end{aligned}
\tag{S1}
$$

We simulated these augmented equations with $n_D = 100$, which is still computationally tractable but results in variation in the disclosure period of less than one month. Then, we compared the impact

www.pnas.org/cgi/doi/10.1073/pnas.1814647116

on bed bug prevalence and the costs to landlords over time after implementation of disclosure policies. We did this for the baseline parameters considered in the main text (Table 1) as well as for a very low prevalence (1%). We found that modeling the disclosure period as nearly-fixed time results in a slight increase in the percent of vacant units over time and hence a slight increase in vacancy (by less than $ 4 per unit per year), as well as a very small decrease in prevalence and hence decrease in treatment cost (by less than $0.5 per unit per year) (Figures S13-S14). Overall, these minor changes made no difference in the conclusions of the paper.

Considering disclosure from the economic perspective of renters

The analysis presented in the main text focused on the economic perspective of landlords as this group is widely considered to be more likely punished by disclosure policies. Given that the primary aim of disclosure policies is to provide protection to renters, we expected renters to benefit financially from disclosure policies. We tested this hypothesis by explicitly considering the economic perspective of renters with the same SIS model (Eq. 2) and baseline parameter values (Table 1) used to assess cost to landlords in the main text. We also assessed costs to renters across a range of baseline bed bug prevalence and renter selectivity values to see how renters are expected to be impacted under these different scenarios.

From the perspective of renters, costs associated with bed bugs can be grouped into one of the following: ancillary treatment costs, moving costs, and cost of untreated infestations. Jurisdictions that adopt disclosure policies often require landlords to pay for all necessary bed bug treatments. However, there are a number of ancillary costs associated with bed bug treatment that renters often have to bear. These may include: replacing infested mattresses, box springs, bed frames, couches, or other furniture that cannot be remediated by extermination; professional laundering of infested bedding and clothing; cleaning infested carpets, curtains or upholstery; buying mattress covers, bed bug monitors, and other items to monitor and prevent re-infestation.

Because we assumed that presence of a bed bug infestation would compel renters to move out of a unit more readily than they would at baseline (parameter b in Eq. 2), moving costs must be included in estimating the economic impact of disclosure to renters. Costs associated with moving out of a unit may include fees to moving companies, rental agents, storage facilities, utilities companies, as well as lost deposits, penalties for breaking lease (in the case of early termination), and time off work needed to move. Finally, the cost of untreated infestations includes all costs incurred so long as an infestation remains untreated. Some of these costs are similar to the costs of ancillary treatment since renters may replace furniture and bedding and/or seek laundering services, as needed, during an infestation and not just to complement bed bug treatment. Cost of untreated infestations can also take the form of missed work as some employers are responding to growing reports of bed bugs in the workplace [S3, S4] by asking employees with infestations in their homes to abstain from work [S5].

The average per-unit number of bed bug treatments occurring in a given year is equal to the number of transitions from infested to susceptible classes for that year divided by the number of rental units in the population N. The average per-unit number of move-out events is equal to the number of transitions from occupied to vacant classes divided by N, and the average time that each unit is infested is equal to the total unit-time spent in infested classes divided by N. Then if c_{anc}, c_{mov}, and c_{inf} are constants equal to the average ancillary cost of bed bug treatment, average cost of moving, and average cost of untreated infestation, respectively, then the component costs of disclosure from the perspective of renters can be expressed for a given year Y by the following:

$$\begin{aligned}\text{Ancillary treatment cost} =& \frac{c_{anc}}{N}\left(\int_{Y}^{(Y+1)}(\gamma I_r+\gamma I_v)\,dt\Big|_{s=s}-\int_{Y}^{(Y+1)}(\gamma I_r+\gamma I_v)\,dt\Big|_{s=0}\right)\\ \text{Moving cost} =& \frac{c_{mov}}{N}\left(\int_{Y}^{(Y+1)}(mS_r+bmI_r)\,dt\Big|_{s=s}-\int_{Y}^{(Y+1)}(mS_r+bmI_r)\,dt\Big|_{s=0}\right)\\ \text{Cost of untreated infestation} =& 12\frac{c_{inf}}{N}\left(\int_{Y}^{(Y+1)}(I_r+I_v)\,dt\Big|_{s=s}-\int_{Y}^{(Y+1)}(I_r+I_v)\,dt\Big|_{s=0}\right)\end{aligned} \tag{S2}$$

In calculating the economic impact of disclosure on renters, we assume that renters form a closed population and all renters are housed at all times. Little information exists on costs associated with bed bugs from the renter's perspective, so we made conservative estimates for all cost constants using available data. We estimated the average ancillary cost of treatment (c_{anc}) to equal \$800, roughly equivalent to the cost of replacing a low- to mid-price queen mattress [S6]. We estimated the average cost of moving (c_{mov}) to equal \$400, the lower range listed for the estimated cost of hiring a moving company for a local move to a 2-bedroom apartment [S7]. Finally, we estimated the cost of untreated infestation (c_{inf}) to be equal to \$200, roughly equivalent to one full day's work (i.e. 8 hours) at the national hourly wage for private employees in 2017 [S8]. While we present results obtained using these cost estimates, readers interested in viewing results for alternate values for bed bug-related costs to renters can visit our R Shiny web application available at https://bedbugdisclosure.shinyapps.io/shinyapp/.

Overall, we found disclosure to benefit renters from the first year of disclosure, with savings that grow over time as bed bug prevalence is reduced (Figure S4). All cost components with respect to renters (i.e. ancillary treatment cost, moving cost, and cost of untreated infestation) were found to decrease with disclosure, reflecting a savings to renters across all categories. Net savings were estimated to average about \$5 per unit in year one of disclosure and grow to about \$120 per unit by year 20, though these were calculated with conservative price estimates of bed bug-related costs and are, thus, likely to underestimate actual benefits to renters. Renters are expected to benefit from disclosure regardless of the baseline bed bug prevalence and renter selectivity (so long as $s>0$), with greatest financial benefit in situations where baseline prevalence renter selectivity is high (Figure S5). These conditions are the same as those in which prevalence is expected to be reduced by the greatest margin (Figure 4), which further supports the interpretation that these benefits are driven by reduction in bed bug prevalence.

Discounting future disclosure costs

In the main text, we present yearly costs that are expected to follow the implementation of a disclosure policy in their raw, unadjusted form. However, some readers may be interested in projected costs and savings that take into account discounting. Discounting is the idea that money received in the future is less valuable than money received in the present, which can be due to concrete considerations like the rate of return on investments or more abstract considerations like risk, uncertainty, or quality of life. We added discounting to our projections by calculating the present value for future costs and savings. Setting the start of a disclosure policy as year 0, we adjusted a future cost C expected in year Y for a discount rate r with the equation:

$$\text{Discounted cost (aka present value)} = \frac{1}{(1+r)^Y}\cdot C \tag{S3}$$

Results are shown for discount rates between 2% and 10% in Figure S3. Overall, we found discounting to very slightly reduce the magnitude of the initial yearly costs (in the first 4 years), and to also partially reduce the eventual expected savings (year 5 and onwards), especially in later years. The year at which costs turned to savings did not perceptibly change (still around year 5), although discounting did slightly delay the point at which cumulative costs converted to savings (from around year 7 with no discounting to around year 9 with 10% discounting). Based on the inflation-adjusted Case-Schiller home price index

[S9, S10], the annual average rate of home appreciation is around 1% nationally, suggesting that in many housing markets the discounting rate is likely to be small.

Effect of inter-market migration

We considered a model that relaxes the assumption of a closed population to determine how a rental market with legislated disclosure policies might be impacted by surrounding markets that do not adopt such policies. We assume that a fraction, i, of new tenants moving into vacant units come from external markets that have a net bed bug prevalence e. These tenants have the same probability of relocation transmission (k) as local tenants. The model with the addition of inter-market migration is given below:

$$\begin{aligned}
\frac{dS_r}{dt} =& -\beta S_r I_r/N + \gamma I_r + n((1-i)(1-kf(t)) + i(1-ke))S_v \\
&+ (1-s)n\left[(1-i)(1-kf(t)) + i(1-ke)\right]S_v' - mS_r \\
\frac{dI_r}{dt} =& \beta S_r I_r/N + nk((1-i)f(t) + ie)S_v + (1-s)nk((1-i)f(t) + ie)S_v' + n(1-s)I_v \\
&- (\gamma + bm)I_r \\
\frac{dS_v}{dt} =& mS_r + (1/D)S_v' - nS_v \\
\frac{dI_v}{dt} =& bmI_r - (\gamma + n(1-s))I_v \\
\frac{dS_v'}{dt} =& \gamma I_v - (n(1-s) + 1/D)S_v' \\
N =& S_r + I_r + S_v + I_v + S_v' \\
f(t) =& \frac{bI_r}{S_r + bI_r}
\end{aligned} \tag{S4}$$

Impact of disclosure in a structured population

We examined how our results depend on the modeling assumption of a uniform and well-mixed rental population by constructing two-population models that represent extreme cases where infestations are concentrated in a detached, minority population. Each model contains a high-prevalence subpopulation that comprise 20% of the total population and can sustain higher levels of infection by having one of the following divergent characteristics with respect to the majority subpopulation: a lower treatment rate γ (average time to treatment of 1 year vs 4 months), a higher move-out rate m (average time in unit of only 1 year vs 5 years, n adjusted to keep vacancy rates constant), or a higher "aversion to bed bugs" (higher b and k, representing likelihood of leaving an infested unit and preventing infestation of a new unit). We assume these two subpopulations are completely disconnected, and so infestation cannot be transferred from units in one subpopulation to those in the other. All other parameters are kept constant between the two subpopulations, and as in the one-population case, the β parameter was estimated to give an overall population prevalence of 5%. The specific parameter combinations we considered and the results for these scenarios are reported in Supplementary Table S1.

In the scenario where treatment rates differ between subpopulations, we found that the epidemic was sustained solely by the high-prevalence subpopulation (with a prevalence of 25% vs 0% in low-prevalence population). When disclosure was implemented we found that costs always remained positive; there were never savings in the long-term. This occurred because prevalence remained high, and savings due to lower infestation rates could not offset vacancy costs. Only if infested units were never rented out after implementation of disclosure ($s = 1$) – an unrealistic scenario which assumes prospective tenants both have leverage and elect to turn down all units with an infestation history – did savings eventually accrue. When the subpopulations differed in their tenant turnover rates, bed bugs were endemic in both groups but at very different prevalences (12% vs 3%). Disclosure resulted in minor costs turning to minor savings in the low-prevalence group, and high costs turning to near-zero costs in the high-prevalence group. The scenario where b and k ("aversion") differ between subpopulations was nearly identical to the first scenario

where treatment rates differed: infestation was again maintained only in the high-prevalence group, and disclosure remained costly.

Model limitations and potential extensions

Metapopulation and network structure

One of the major assumptions of our model is that the rental market is a homogeneous and well-mixed population, where each unit in the population has the same rates of being rented and vacated, and is equally likely to become infested or transfer infestation to other units. In reality, the rental market is likely to be more heterogeneous, with rates of tenant turnover, infestation, and treatment differing across subpopulations. We examined the effect of relaxing the assumption of homogeneous mixing by analyzing the effects of disclosure in a two-population model where the subpopulations differed with respect to these parameters. However, we did not consider more complex population structures because it is not clear, given the lack of data on bed bug transmission dynamics, which level of population structure would be most appropriate to include. Here, we present a few possibilities for the inclusion of more detailed population structure that may be pursued as more information on transmission dynamics becomes available.

The model can be extended in future studies to incorporate metapopulation structure to capture spatial heterogeneity in the transmission process, which may result if people tend to move and transmit more often within vs. between neighborhoods, or, if bed bugs themselves move more readily within buildings (or adjacent buildings) than between them. Studies of infestation in multi-unit apartment buildings have found that infestations do, indeed, spread readily between adjacent units [S11, S12, S13]. However, these results may depend on building type and spatial scale: a survey of bed bug infestations conducted in a census tract comprising row homes (in which adjacent homes share a wall) found infestations to have low spatial autocorrelation [S14]. Network structure could also be incorporated to capture demographic heterogeneity in the transmission process. To date, no study has examined the spread of bed bugs along social networks, but social contacts are likely to play a role in the transmission process as bed bugs are transmitted on human possessions, which are more likely to be exchanged between friends and family members.

Market elasticities and price dynamics

Our model does not include market elasticities, so it cannot capture the behaviors of landlords who might elect to decrease rent to increase the desirability of units affected by disclosure nor the associated downstream effects. In the main text, we discuss how these behaviors could lead us to overestimate vacancy costs and the amount of prevalence reduction. Downward pressure on the price of units with an infestation history may also result in a reciprocal premium on uninfested units. A division may thus emerge in the rental market: infested and recently infested units that are affordable to lower income tenants and uninfected units that are not. Such an outcome is a concerning possibility, but certainly not inevitable. Still, municipalities adopting disclosure laws might pursue complementary programs that ensure an adequate supply of quality, affordable home. Future modeling work could combine our population dynamic approach with more detailed economic models to study this issue in more detail.

In addition, our forecasts for costs to landlords over time also assume that none of the underlying parameters of our model change during the timescale considered. However, it is possible that the relative costs of vacancy and turnover could change with demographic or economic shifts in a region and that time of effective treatment and cost of that treatment could change based on pest control practices, resistance of bed bug populations, or public awareness of bed bugs.

Landlord compliance

Our model assumes that landlords would comply perfectly with disclosure laws. Imperfect compliance would lead to effective values of renter selectivity (s) and the disclosure period (D) that are smaller than what is specified by the model; with less disclosure, tenants would be more likely to rent an infested apartment, and fewer infested units would move to the quarantined class S'_v. The results in Figures 3 and 4 show that imperfect compliance (lower s) would result in less initial costs, less long-term savings, and a lower reduction in prevalence than perfect compliance. Another potential effect of imperfect

compliance is that disclosure would penalize compliant landlords with vacancy costs, while the savings due to decreased prevalence would indiscriminately benefit all (compliant and non-compliant) landlords. This highlights the need for strong enforcement of disclosure policies to achieve optimal outcomes.

Supplementary Tables

Table S1: **Parameters and outputs from simple two-population models**. All parameters are at the values given in Table 1 unless explicitly stated. The first subpopulation always comprises 80% of the total rental unit population, and the second 20%.

			Differences between subpopulations		
	None		Treatment rates	Tenant turnover rates	Aversion to bed bugs
Overall prevalence	5%		5%	5%	5%
β (yr^{-1})	2.29		1.38	2.32	2.46
Parameters		1	$\gamma_1 = 3\,\text{yr}^{-1}$	$m_1 = 0.2\,\text{yr}^{-1}$, $n_1 = 2.4\,\text{yr}^{-1}$	$b_1 = 5$, $k_1 = 0.1$
		2	$\gamma_2 = 1\,\text{yr}^{-1}$	$m_2 = 1\,\text{yr}^{-1}$, $n_2 = 12\,\text{yr}^{-1}$	$b_2 = 1.3$, $k_2 = 1$
Baseline prevalence	5%	1	0	3%	0
		2	25%	12%	25%
R_0	1.05	1	0.45	1.03	0.95
		2	1.34	2.1	1.33
Peak cost of disclosure (per unit)	$27	1	0	$16	0
		2	$192	$82	$161
Net cost 10 years after disclosure (per unit)	-$37	1	0	-$5	0
		2	$118	$3	$110
Final prevalence (~100 years post-disclosure)	1%	1	0	1%	0
		2	20%	6%	22%

Supplementary Figures

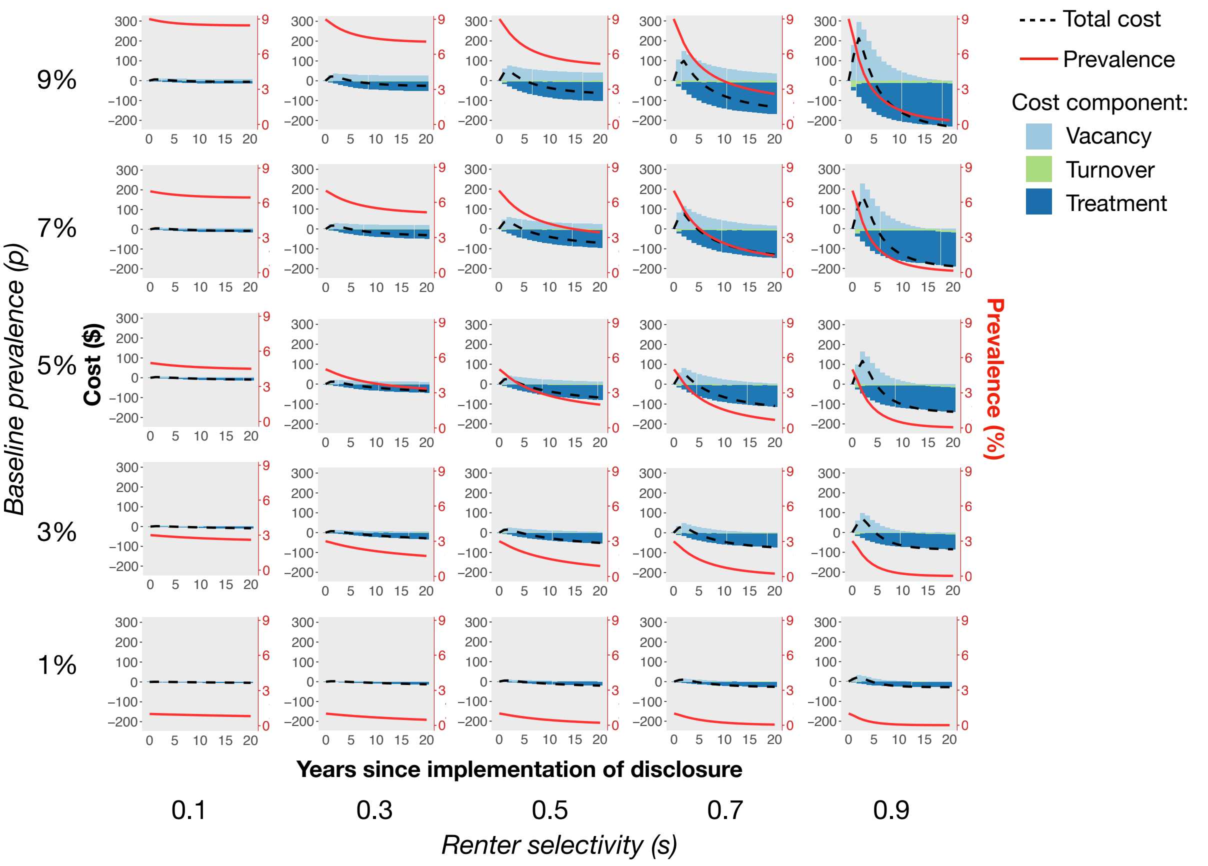

Figure S1: **Costs and benefits of disclosure under different scenarios for baseline prevalence of infestation and impact of disclosure on renter preference.**

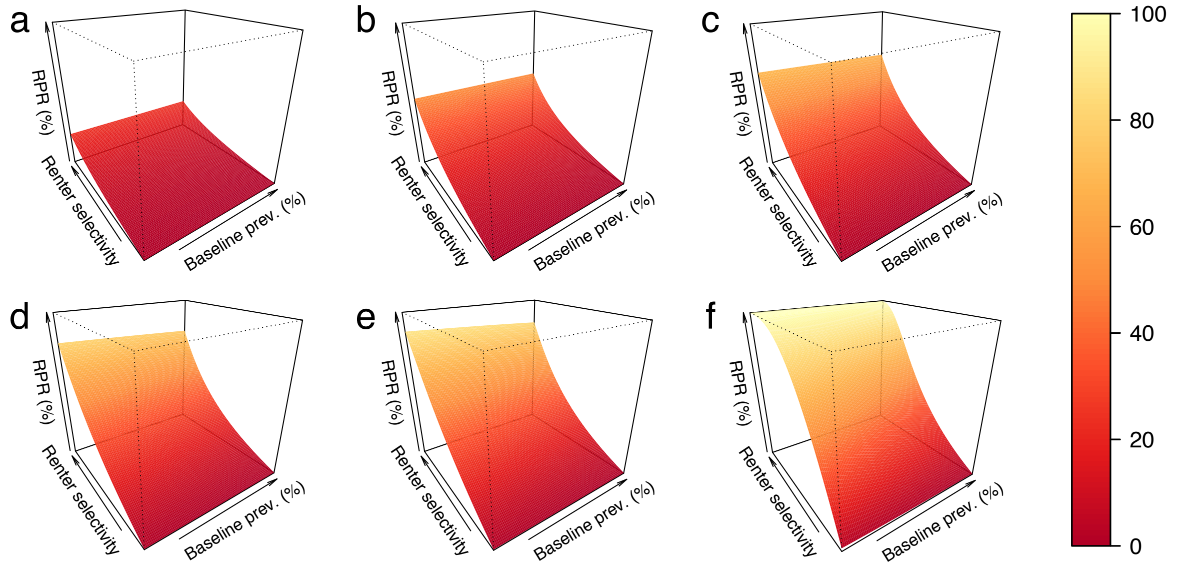

Figure S2: **Relative prevalence reduction (RPR) of bed bug infestations over time after implementation of disclosure, as a function of the baseline prevalence and renter selectivity.** Results are presented for years 1 (a), 2 (b), 3 (c), 4 (d), 5 (e), and 20 (f) after the implementation of a disclosure policy. Baseline prevalence (p) ranges from 0.1 to 10% and renter selectivity s ranges from 0.01 to 1.

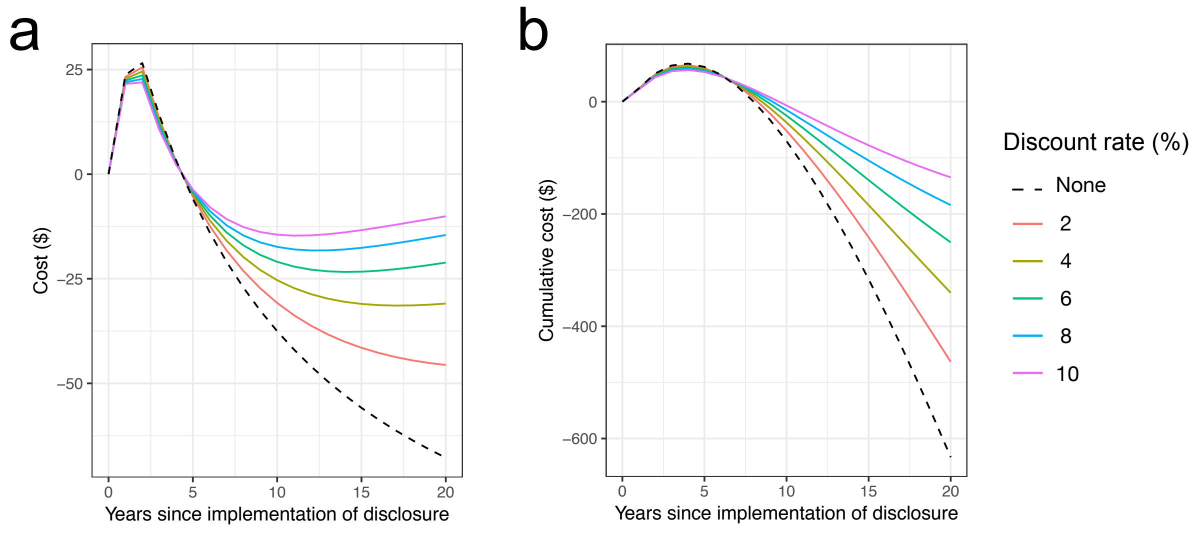

Figure S3: **Yearly (a) and cumulative (b) costs of disclosure under different discount rates**

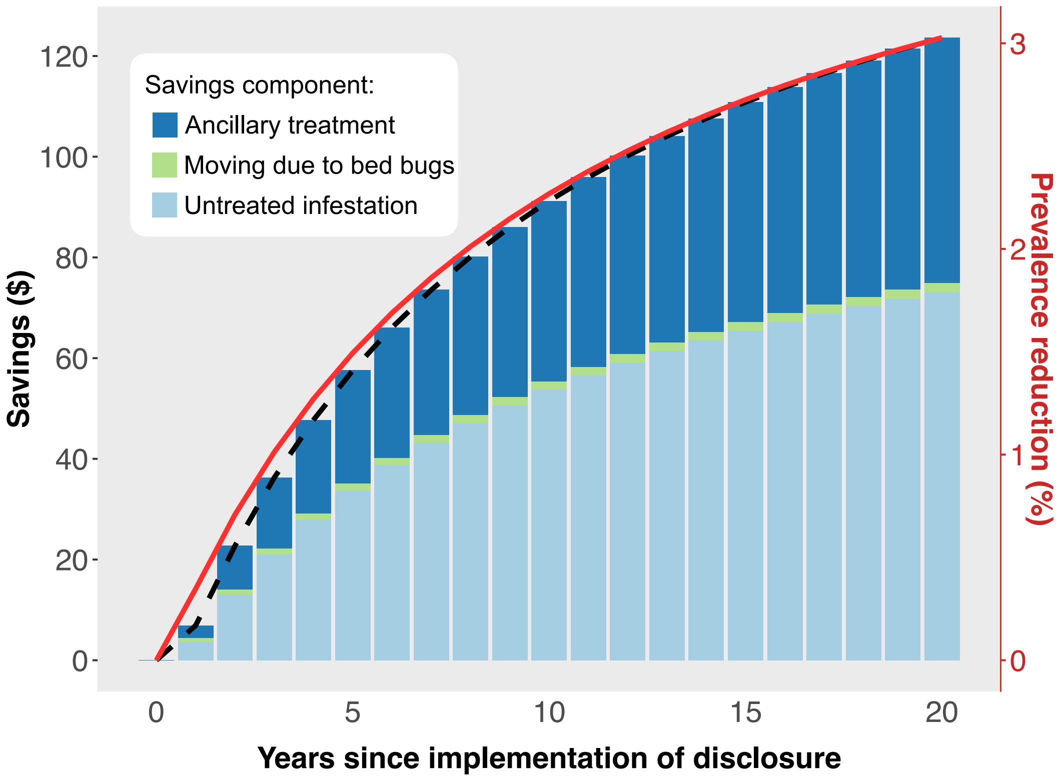

Figure S4: **Impact of disclosure from the economic perspective of renters.** The overall savings to renters, defined as the difference in cost to renters in the presence of disclosure compared to no disclosure, is shown by the dashed black line as a function of time since a disclosure policy is implemented. Components of cost savings are also shown as bars representing averages over one-year periods. The term *savings* reflect the fact that costs are lower in the presence of disclosure compared to no disclosure for all cost components at all years after disclosure is implemented. The overall *reduction* in the prevalence of infestations in the population is shown by the red line. The model was run by assuming that before the implementation of disclosure, the baseline prevalence of infestations was at a steady state value of 5% and that disclosure discouraged but did not prevent rental of disclosed units ($s = 0.5$). Results for other parameter combinations are shown in Figure S5.

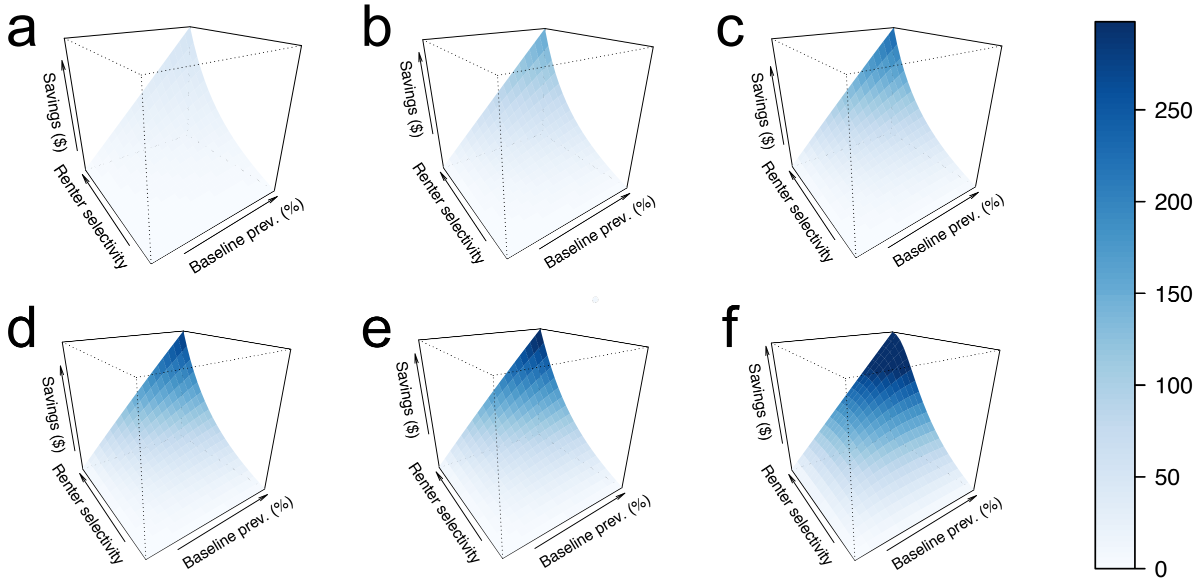

Figure S5: **Total per-unit savings due to disclosure from the perspective of renters, as a function of the baseline prevalence and renter selectivity.** Results are presented for years 1 (a), 2 (b), 3 (c), 4 (d), 5 (e), and 20 (f) after the implementation of a disclosure policy. Darker blue colors represent parameter regimes and time-points where savings to renters due to disclosure are highest. An animation showing the dependence of savings to renters on p and s over the initial twenty years of disclosure is available at https://bedbugdisclosure.shinyapps.io/shinyapp/.

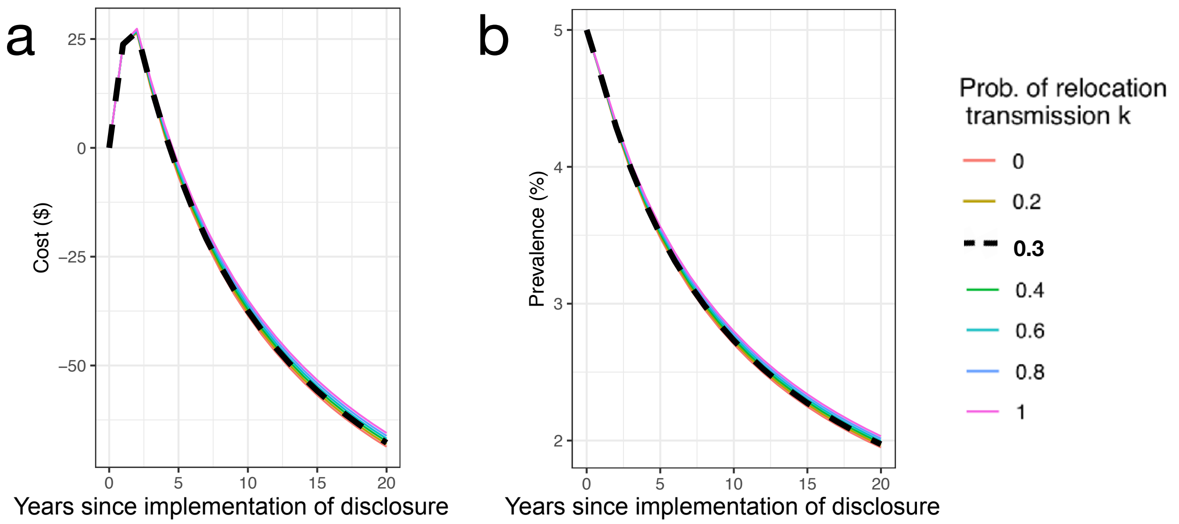

Figure S6: **Sensitivity of model predictions for the impact of disclosure policies to the relocation transmission rate (k).** The vacancy multiplier b is set to the baseline value of 1.3.

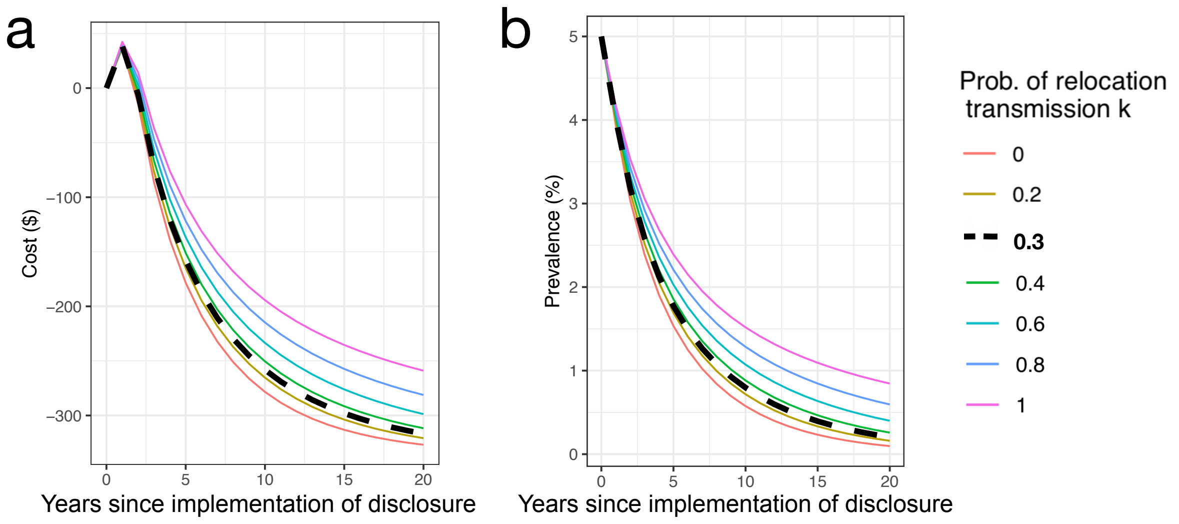

Figure S7: **Sensitivity of model predictions for the impact of disclosure policies to the relocation transmission rate (k).** The vacancy multiplier b is set to 5.

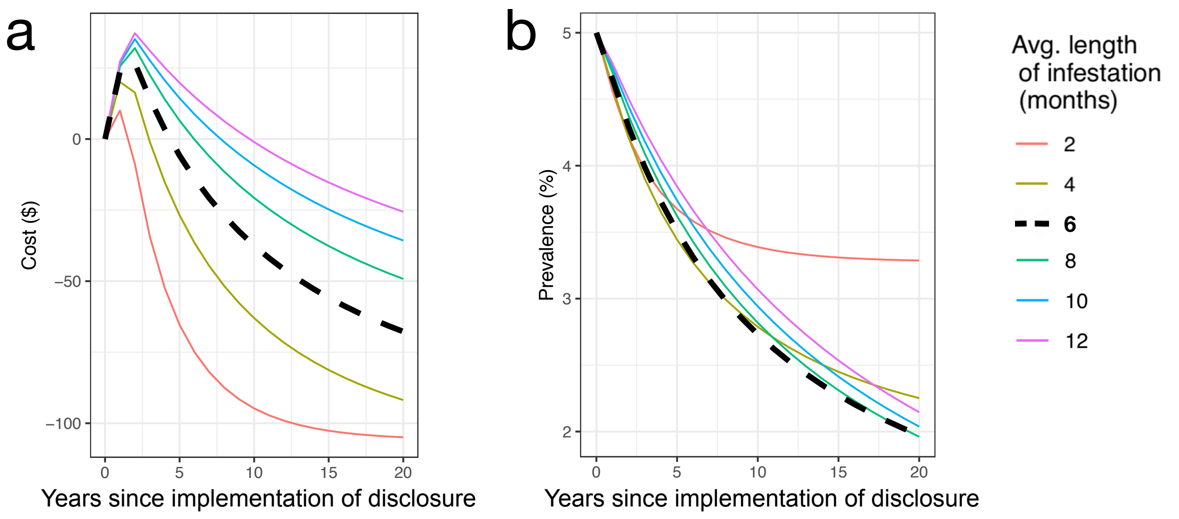

Figure S8: **Sensitivity of model predictions for the impact of disclosure policies to the treatment rate ($1/\gamma$).**

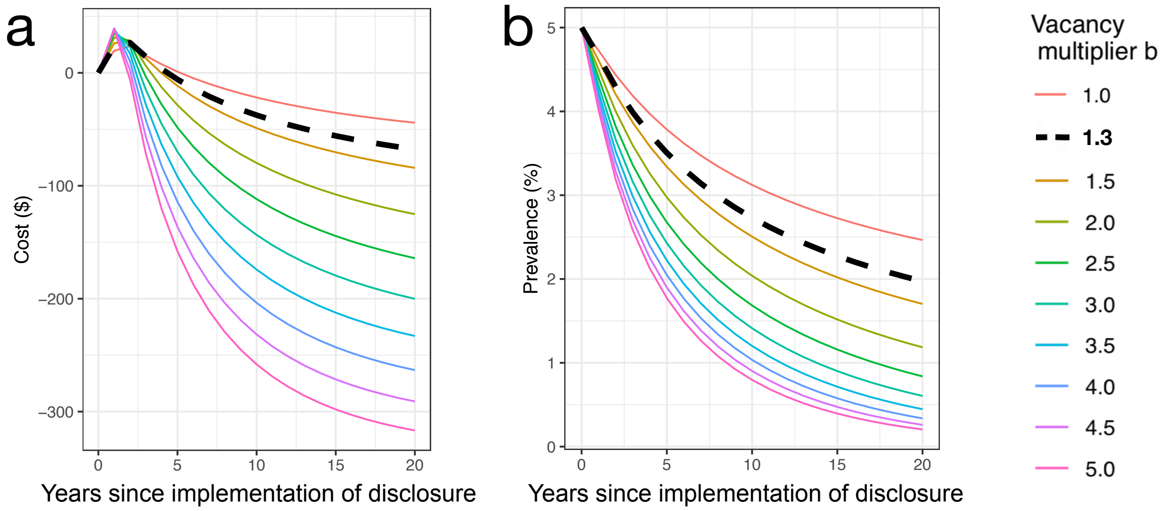

Figure S9: **Sensitivity of model predictions for the impact of disclosure policies to the vacancy multiplier (b).**

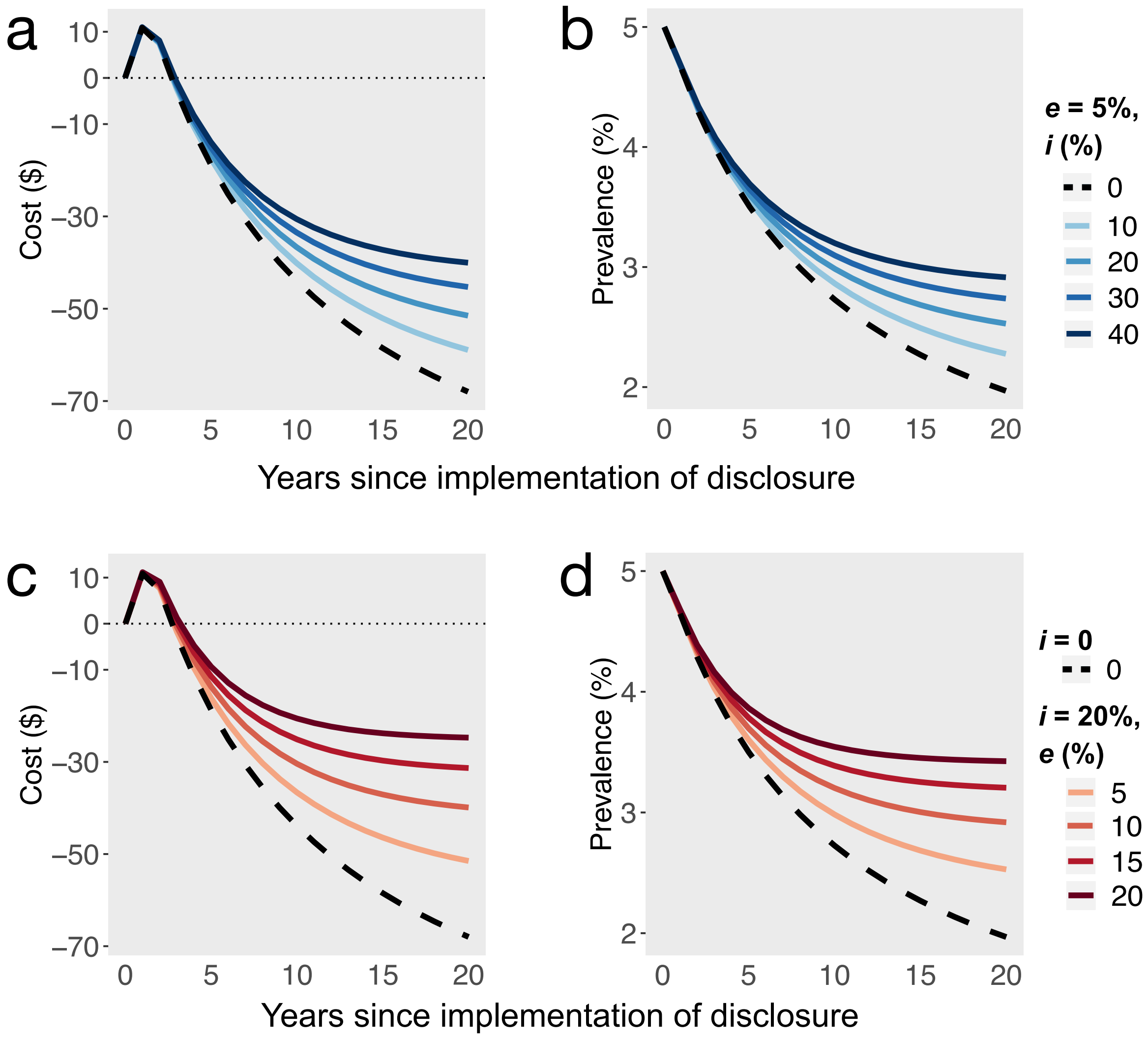

Figure S10: **Impact of inter-market migration on disclosure cost and bed bug prevalence**. Cost to landlords (a) and bed bug prevalence (b) for the twenty years after implementation of disclosure when tenants from an external market with a 5% bed bug prevalence make up 10 to 40% of new move-ins are represented by solid lines, with darker blue representing higher immigrant fractions. Overall cost (c) and bed bug prevalence (d) for the twenty years after implementation of disclosure when 20% of new tenants come from external markets and the bed bug prevalence of the external market, e, is varied from 5 to 20% are represented by solid lines, with darker red representing higher external bed bug prevalence. The reference case (i.e. when rental units form a closed population and $i = 0$) is represented in all panels by black dashed lines. An R Shiny web application that allows users to input alternate values for i and e and visualize prevalence and cost (including cost components) is available at https://bedbugdisclosure.shinyapps.io/shinyapp/.

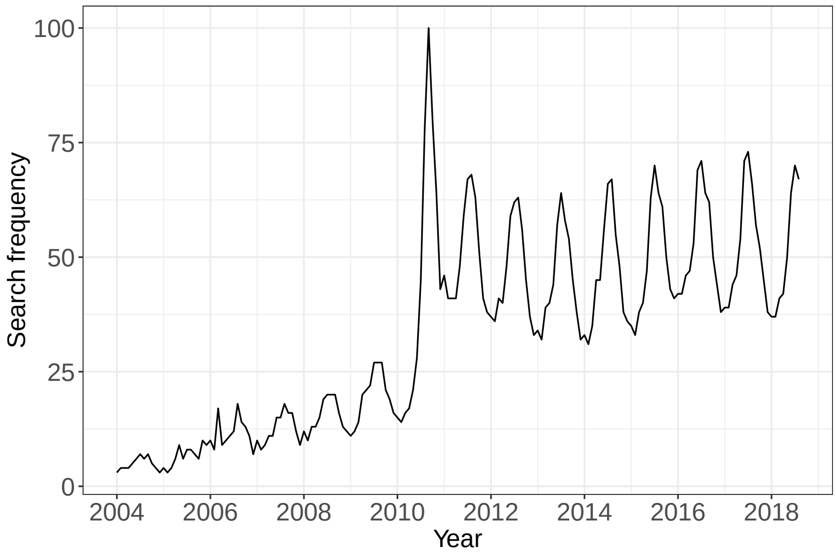

Figure S11: **Google search frequency for the phrase "bed bugs" in the United States from January 2004 to August 2018.** Search frequency is normalized so that peak frequency is equal to 100. In addition to exhibiting seasonal oscillations (which correspond to reports that bed bug complaints and calls to exterminators peak in summer months [S15, S3]), bed bug queries have increased annually from 2004 until they reached a plateau in 2012, suggesting that the prevalence of bed bug infestations has remained relatively stable for several years. Data is available from Google Trends at `https://trends.google.com/trends/`.

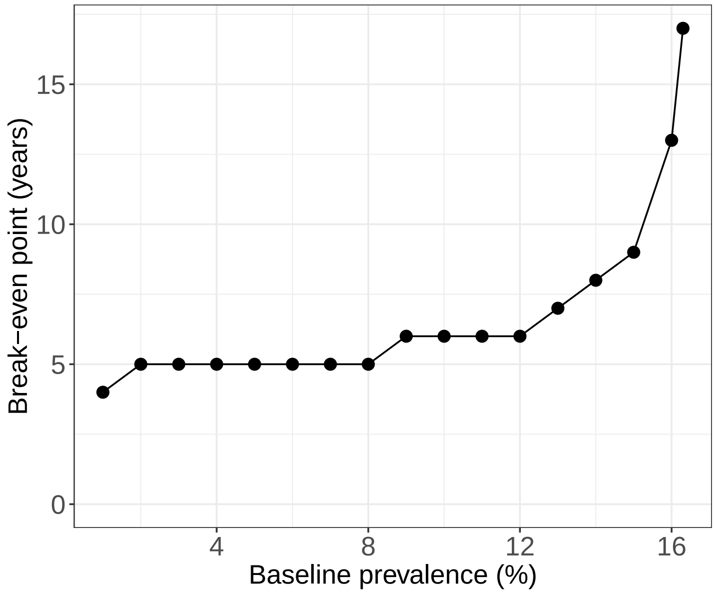

Figure S12: **The dependence of break-even point (i.e. time-point at which disclosure costs become savings) to baseline prevalence.**

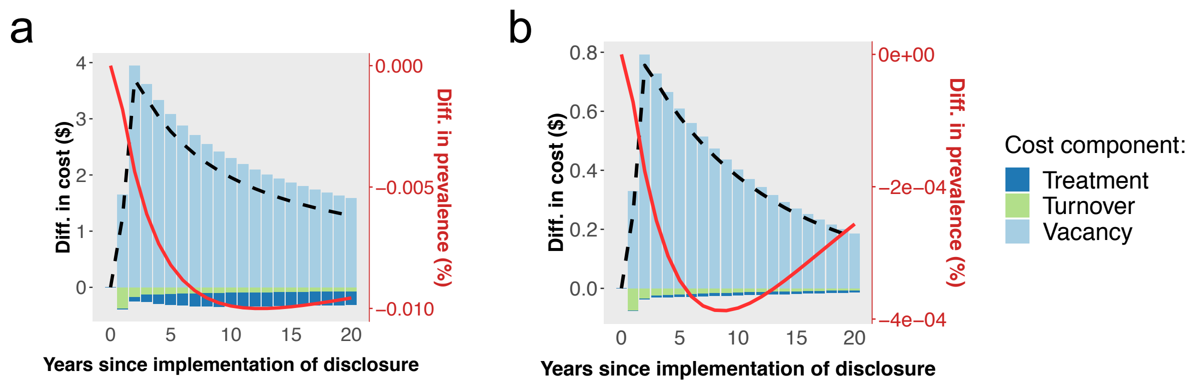

Figure S13: **Difference in costs and prevalence calculated from the disclosure delay model compared to the basic disclosure model for a starting baseline prevalence of 5% (a) and 1% (b).** All other parameter values were set equal to the baseline values given in Table 1.

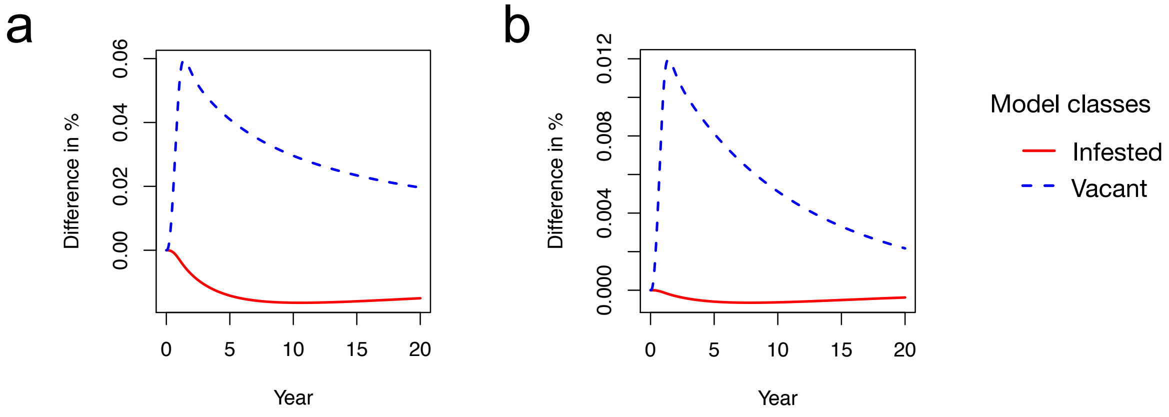

Figure S14: **Difference in the percentage of infested and vacant units calculated from the disclosure delay model compared to the basic disclosure model for a starting baseline prevalence of 5% (a) and 1% (b).** Infested classes include I_r and I_v while vacant classes include S_v, I_v, and all S'_v classes. All other parameter values were set equal to the baseline values given in Table 1.

SI References

[S1] Iulia A. Neagu, Jason Olejarz, Mark Freeman, Daniel I. S. Rosenbloom, Martin A. Nowak, and Alison L. Hill. Life cycle synchronization is a viral drug resistance mechanism. *PLOS Computational Biology*, 14(2):e1005947, February 2018.

[S2] Daniel L. Floyd, Stephen C. Harrison, and Antoine M. van Oijen. Analysis of Kinetic Intermediates in Single-Particle Dwell-Time Distributions. *Biophysical Journal*, 99(2):360–366, July 2010.

[S3] Michael F Potter, Kenneth F. Haynes, and Jim Fredericks. Bed bugs across America: The 2015 Bed Bugs Without Borders Survey. *PestWorld*, page 4–14, 2015.

[S4] Jane A Gwira Baumblatt, John R Dunn, William Schaffner, Abelardo C Moncayo, Annica Stull-Lane, and Timothy F Jones. An outbreak of bed bug infestation in an office building. *Journal of Environmental Health*, 76(8):16–19, 2014.

[S5] Dana Wilkie and Allen Smith. What to Do When Bedbugs Bite at Work, June 2017.

[S6] The Mattress Price Comparison You Can Actually Trust - 2018. *Sleep Like the Dead*, 2018. Available at http://www.sleeplikethedead.com/mattress-compare-price.html. Accessed: 3 December 2018.

[S7] Mary Boone. How Much Does it Cost to Move? *Zillow Porchlight*, 2018. Available at https://www.zillow.com/blog/how-much-will-you-pay-to-move-91663/. Accessed: 3 December 2018.

[S8] Bureau of Labor Statistics. Table B-3. average hourly and weekly earnings of all employees on private nonfarm payrolls by industry sector, seasonally adjusted. *Economic News Release*, 2018. Available at https://www.bls.gov/news.release/empsit.t19.htm#ces_table3.f.p. Accessed: 3 December 2018.

[S9] Robert J. Shiller. *Irrational Exuberance*. Crown Business, New York, 2 edition edition, May 2006.

[S10] Standard & Poor. Case-Shiller Home Price Indices Indices, 2018.

[S11] Changlu Wang, Kurt Saltzmann, Eva Chin, Gary W Bennett, and Timothy Gibb. Characteristics of cimex lectularius (hemiptera: Cimicidae), infestation and dispersal in a high-rise apartment building. *Journal of Economic Entomology*, 103(1):172–177, 2010.

[S12] Warren Booth, Virna L Saenz, Richard G Santangelo, Changlu Wang, Coby Schal, and Edward L Vargo. Molecular markers reveal infestation dynamics of the bed bug (hemiptera: Cimicidae) within apartment buildings. *Journal of Medical Entomology*, 49(3):535–546, 2012.

[S13] Richard Cooper, Changlu Wang, and Narinderpal Singh. Mark-release-recapture reveals extensive movement of bed bugs (cimex lectularius l.) within and between apartments. *PLoS One*, 10(9):e0136462, 2015.

[S14] Yage Wu, Dylan M Tracy, Alexis M Barbarin, Corentin M Barbu, and Michael Z Levy. A door-to-door survey of bed bug (Cimex lectularius) infestations in row homes in Philadelphia, Pennsylvania. *The American Journal of Tropical Medicine and Hygiene*, 91(1):206–210, 2014.

[S15] Tarub S Mabud, Alexis M Barbarin, Corentin M Barbu, Katelyn H Levy, Jason Edinger, and Michael Z Levy. Spatial and temporal patterns in cimex lectularius (hemiptera: Cimicidae) reporting in philadelphia, pa. *Journal of Medical Entomology*, 51(1):50–54, 2014.
